# Comparison of clinical outcomes among cancer patients treated in and out of clinical trials

**DOI:** 10.1186/s12885-023-11305-3

**Published:** 2023-08-23

**Authors:** Jose A. Carreno Duenas, Natalia Sanchez P., Carlos E. Bonilla

**Affiliations:** 1 Instituto Nacional de Cancerología - Colombia, Calle 1 # 9-85, Bogota, 110111 Colombia; 2 Centro de tratamiento e investigación sobre cáncer (CTIC), Calle 168 # 14 -49, Bogota, 110131 Colombia

## Abstract

**Background:**

It is unknown if participation in a cancer clinical trial confers clinical benefits to patients. There is not enough scientific evidence in this regard and the available publications are scarce and provide ambiguous and limited information.

**Objective:**

Compare overall and progression-free survival and response to treatment among those who met the eligibility criteria and accepted to participate and those who refused to participate in cancer clinical trials.

**Methods:**

An observational cross-sectional study with an analytical component was carried out, which included patients diagnosed with cancer who participated in phase III clinical trials and patients who, being eligible, refused to participate. The patients were cared for at the National Institute of Cancerology in Colombia between 2019 and 2022. Analysis of differences in proportions and means of sociodemographic and clinical variables was included; overall survival and progression-free survival time were described and the survival curves between groups were compared. Variables related to survival were determined using a Cox regression model and Hazard Ratios were calculated.

**Results:**

62 women and 50 men were included. In the women group, we found a statistical association between clinical trial participation and non-serious events adverse and progression. The stable disease and complete response were higher in participants than in refusers. The median progression-free survival for refusers was 7,4 m meantime for participants the median was not reached and 74,1% remained without progression at 28 months. In the men group, we also found a statistical association between clinical trial participation and the occurrence of non-serious events adverse meanwhile there were no significant differences in overall response, progression, and death, even though the proportion of progression was minor in participants 20% vs. refusers 26% respectively. The median survival was not reached for any group, even though in the participants group 55,2% were still alive at month 20 and in the refusers group still alive at 56,8% at month 45. Covariables included for the multivariate Cox regression only age had a statistical association with overall survival in the women’s group and the men group any covariables reached statistical association.

**Conclusion:**

It can be considered that participation in clinical trials could give participants a better response to treatment, without increasing the probability of death and with the probability of decreasing the progression of the disease. Participation in trials could improve the outcomes of clinical response rates, no change in overall survival, and progression-free.

## Introduction

Clinical trials can provide high evidence of the efficacy and safety of new treatments for the treatment of diseases [1] and are required by regulatory agencies for the approval of new drugs, which is conditional on the impact they produce on clinical outcomes. and the quality of life of patients [2].

The gold standard for measuring the efficacy of anticancer drugs, especially in cancer patients with an advanced or metastatic stage, is overall survival (OS) but takes a long time, and additionally, losses to follow-up or censorship of patients make it difficult to measure. Currently, many new drugs are approved based on the improvement of surrogate outcomes that are measured more quickly such as progression-free survival (PFS), response to treatment, and improvement in the patient’s quality of life [3] which, together with a palliative benefit in reducing cancer-related symptoms, are elements considered essential for a beneficial therapy for cancer patients [4].

Oncological outcomes that are evaluated in cancer clinical trials are classified as primary when they are patient-centered and include, among others, response to treatment, survival time, and those related to quality of life. Secondary outcomes focus on tumor behavior and its response to treatment, which is assessed through diagnostic imaging or biomarker measurement. The time to evaluate the tumor response generally varies between 90 and 120 days after the start of treatment. Composite outcomes between survival, progression and recurrence are reported in some trials [5].

During a clinical trial, the participants have the possibility of receiving investigational treatments under a rigorous follow-up of their condition; Therefore, it is assumed that the partipants would obtain better clinical outcomes. There is even a wide perception among doctors and nurses that clinical trials confer benefits to the participants that are superior to those of regular care [6]. However, the scientific evidence supporting this claim is scant, weak, and inconclusive [6, 7]. Some studies have reported better clinical outcomes in clinical trial participants than in regular care patients; but these results may not be valid due to a lack of comparability and clear differences between groups, because clinical trial participants must meet previously established eligibility criteria that exclude those with diminished general conditions, greater comorbidities, and poor health functional status [8]. It is also possible that those who presented better clinical outcomes were only those assigned to the intervention arm or that they were influenced by psychological aspects such as those presented with the placebo effect, the Hawthorne effect and the “trial effect” [9, 10][11, 12]. We try to make a better comparison of clinical outcomes between the groups, this study included patients who met eligibility criteria to participate in a clinical trial and discriminated between those who agreed to participate and those who refused.

## Material and method

### Patients

An observational cross-sectional study with an analytical component was carried out, a sample size was estimated to establish differences in overall survival and response to treatment in patients diagnosed with locally advanced or metastatic cervical cancer and prostate cancer metastatic patients who participated in phase III clinical trials and in patients who, despite having been eligible, refused to participate. The clinical trials were ongoing and the patients were cared for at the National Institute of Cancerology in Colombia between 2019 and 2022. Cases that presented loss of information were excluded.

This research complied with the guidelines established by the Declaration of Helsinki and the ethical guidelines for biomedical research prepared by the Council for International Organizations of Medical Sciences (CIOMS) and with the parameters established by national regulations, additionally it was approved by the ethic and research committee at the National Cancer Institute. According to the local regulatory frame, it was an investigation without risk, therefore informed consent was not necessary and the results do not contain any data of identification for patients. This research was monitored by an independent monitoring team that verified the validity of the data and information recorded in RedCap (Research Electronic Data Capture).

### Statistical analysis

Descriptive statistical methods were used for sociodemographic, clinical and treatment characteristics, absolute and relative frequencies were estimated for categorical variables, measures of central tendency and dispersion were calculated for quantitative variables; Analyzes stratified by participation in clinical trials, type of cancer, and clinical outcomes were used. Tests were used to determine normality and homogeneity of variances. To determine the difference in proportions between groups, the Xi-squared test or Fisher’s exact test was used, and the Student’s T test was used to determine the difference in means. Overall survival (OS) and progression-free (PFS) time was described graphically and with time-to-event functions, estimated using the Kaplan-Meier method, the survival function and the 95% CI were estimated for the endpoints of time to event. Vital status was verified quarterly for 24 months. Censored data were considered when the follow-up time of a patient ends before death or before completing the observation period, when survival times cannot be accurately established, or when the patient dies from causes unrelated to the event. of interest.

Progression-free and overall survival time was determined from the start date of treatment or the date of randomization, until disease progression or death from any cause. Objective response was assessed using the Response Evaluation Criteria In Solid Tumors (RECIST 1.1) criteria to determine reduction and/or disappearance of tumor size after treatment. Clinical outcomes of each patient were made quarterly up to an observation period of 18 months or until death.

An exploratory analysis was performed for each sex with the exposure variable (participation in clinical trials) and some explanatory covariates such as age, tumor stage, metastatic disease, and ECOG. Survival curves were compared and differences between groups were estimated using the log-rank test. To assess the association between participation and survival, a univariate Cox proportional hazards model was used with the calculation of hazard ratios (HRs) and 95% CI. A multivariable model would be used to assess the prognostic value with those covariates that have demonstrated a level of significance with p values less than or equal to 0.05. Stata 17 was used for data analysis.

## Results and discussion

### Patients

112 patients were included, 51 participants (45.5%) and 61 (54.4%) refusers who were eligible to participate in phase III clinical trials, 62 (55.4%) were women and 50 (44.6%) men. In the women group, the condition was High-risk, locally advanced cervical cancer, and in the clinical trial treatment could include cisplatin chemoradiotherapy with or without Pembrolizumab, meantime in the men group the condition was metastatic castration-resistant prostate cancer the treatment could include Pembrolizumab plus docetaxel versus placebo plus docetaxel. The median age of the participants was 64 years (IQR 30) vs. 61 years (IQR 32) in refusers, 8 women and 16 men participating were older than 65 years. 38.3% of the patients belonged to the subsidized social security system, 93.7% corresponded to socioeconomic strate 1, 2 and 3 and 91.9% had an educational level between primary and secondary. Within the clinical characteristics, 95.5% had a favorable functional status determined by ECOG 1 and 2, the clinical status of the patients was mostly advanced, in stage I and II: 14.2%, stage III: 42.8% and IV: 42.8% (Cervical cancer stage IVA and IVB were included as IV), with lymph node involvement 56.2% and metastatic state 27.6%. The sociodemographic and clinical characteristics are summarized in Table 1.

### Association between clinical outcomes and participation in cancer clinical trials

We found a statistical association between women clinical trial participation and three factors: non-serious events adverse (OR 4,8 IC95% 3,7 − 5,0), progression (OR 0.2 IC95% 0,07 − 0,7), and overall response (OR 5,2 (IC95% 2,3–11,8); the proportion of women with progression disease and partial response was higher in refusers than participants 25,8% vs. 22,5% and 0% vs. 6,4% respectively, meanwhile, stable disease and complete response were higher in participants than refusers 14,5% vs. 8,06% and 12,9% vs. 9,6% respectively. There were no differences between women participants in deaths however the proportion of deaths in participants was minor 9,6% vs. 17,7% respectively (Table 2). On the other hand, in the group of men participants, we also found a statistical association between clinical trial participation and the occurrence of non-serious events adverse (OR 4,2 (IC95% 4,2–7,2) meanwhile there were no significant differences in overall response, progression, and death, even though the proportion of progression was minor in participants 20% vs. refusers 26% respectively (Table 3).

**Table 1:** Patient and tumor characteristics of 112 patients, divided into the clinical trials participants

		Clinical Trials Participants			
		Yes	No	Total	*p* values
**Age**	> 65	24	28	52	0,9
	< 65	27	33	60	
**Sex**	Women	31	31	62	0,29
	Men	20	30	50	
**Location**	Rural	6	6	12	0,74
	Urban	45	55	100	
**Social Security**	Contribute	42	27	69	0,000
	Subsidiade	9	34	43	
**Poverty Level**	1	5	13	18	0,006
	2	11	8	19	
	3	28	40	68	
	4	7	0	7	
**Education Level**	None	0	1	1	0,04
	Primary School	23	23	46	
	High School	22	35	57	
	Technician	3	0	3	
	University	3	0	3	
	Postgraduate	0	2	2	
**Tumor**	Cervix	31	31	62	0,29
	Prostate	20	30	50	
**ECOG**	0	14	26	40	0,08
	1	36	31	67	
	2	1	4	5	
**Clinical Stage**	I	0	8	8	0,000
	II	0	8	8	
	IIIA	2	9	11	
	IIIB	22	15	37	
	IV (IVA – IVB)	27	21	48	
**Clinical T stage**	cT0	9	17	26	0,25
	cT1	9	14	23	
	cT2	3	5	8	
	cT3	15	6	21	
	cT4	11	13	24	
	cTx	3	5	8	
**Clinical N stage**	cN0	20	26	46	0,3
	cN1	16	10	26	
	cN2	1	0	1	
	cN3	0	1	1	
	cNx	13	22	35	
	ND	1	2	3	
**Metastastic disease**	M0	32	44	76	0,47
	M1	17	14	31	
	ND	2	3	5	

**Table 2:** Odds ratios for Clinical Outcomes, comparing women clinical trials participants

		Women Clinical Trials Participant				
**Outcomes**		**Yes**	**No**	**Total**	**OR IC-95%**	*p* values
**Non serious events adverse**	Yes	30	2	32	4,8 (3,7 − 5.0)	**0.000**
	No	1	29	30		
**Overall Response**	PD	14	16	30	5,2 (2,3–11,8)	**0.000**
	SD	9	5	14		
	CR	8	6	14		
	PR	0	4	4		
**Progression**	Yes	7	17	24	0,2 (0,07 − 0,7)	**0.011**
	No	24	14	38		
**Death**	Yes	6	11	17	0,4 (0,13 − 1,3)	0.16
	No	25	20	45		

Overall Response: Progression Disease (PD) - Stable Disease (SD) - Complete Response (CR) - Partial Response (PR)

**Table 3:** Odds ratios for Clinical Outcomes, comparing Men clinical trials participants

		Men Clinical Trials Participant				
**Outcomes**		**Yes**	**No**	**Total**	**OR IC-95%**	*p* values
**Event adverse**	Yes	15	2	17	4,2 (4,3–7,2)	**0.000**
	No	5	28	33		
**Overall Response**	PD	8	14	22	0,7 (0,6-4.2)	0,17
	SD	11	16	27		
	CR	0	0	0		
	PR	1	0	1		
**Progression**	Yes	10	13	23	1,3 (0,4–4,0)	0,64
	No	10	17	27		
**Death**	Yes	6	7	13	1,4 (0,3–5,3)	0.59
	No	14	23	37		

Overall Response: Progression Disease (PD) - Stable Disease (SD) - Complete Response (CR) - Partial Response (PR)

### Disease free survival

**Fig. 1:**
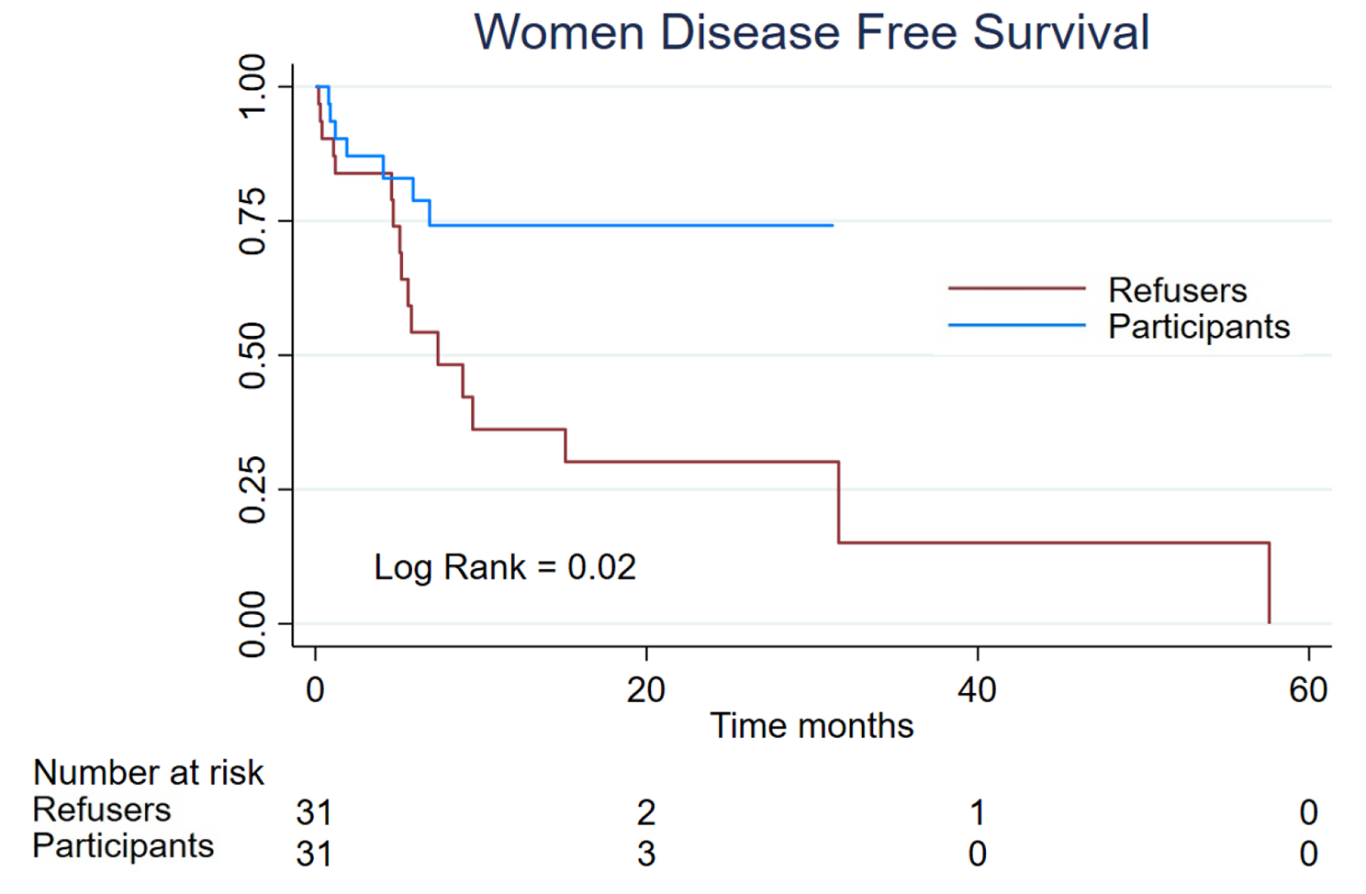
Women Disease Free Survival of 62 patients, divided into the participants (n = 31) and refusers (n = 31)

**Fig. 2:**
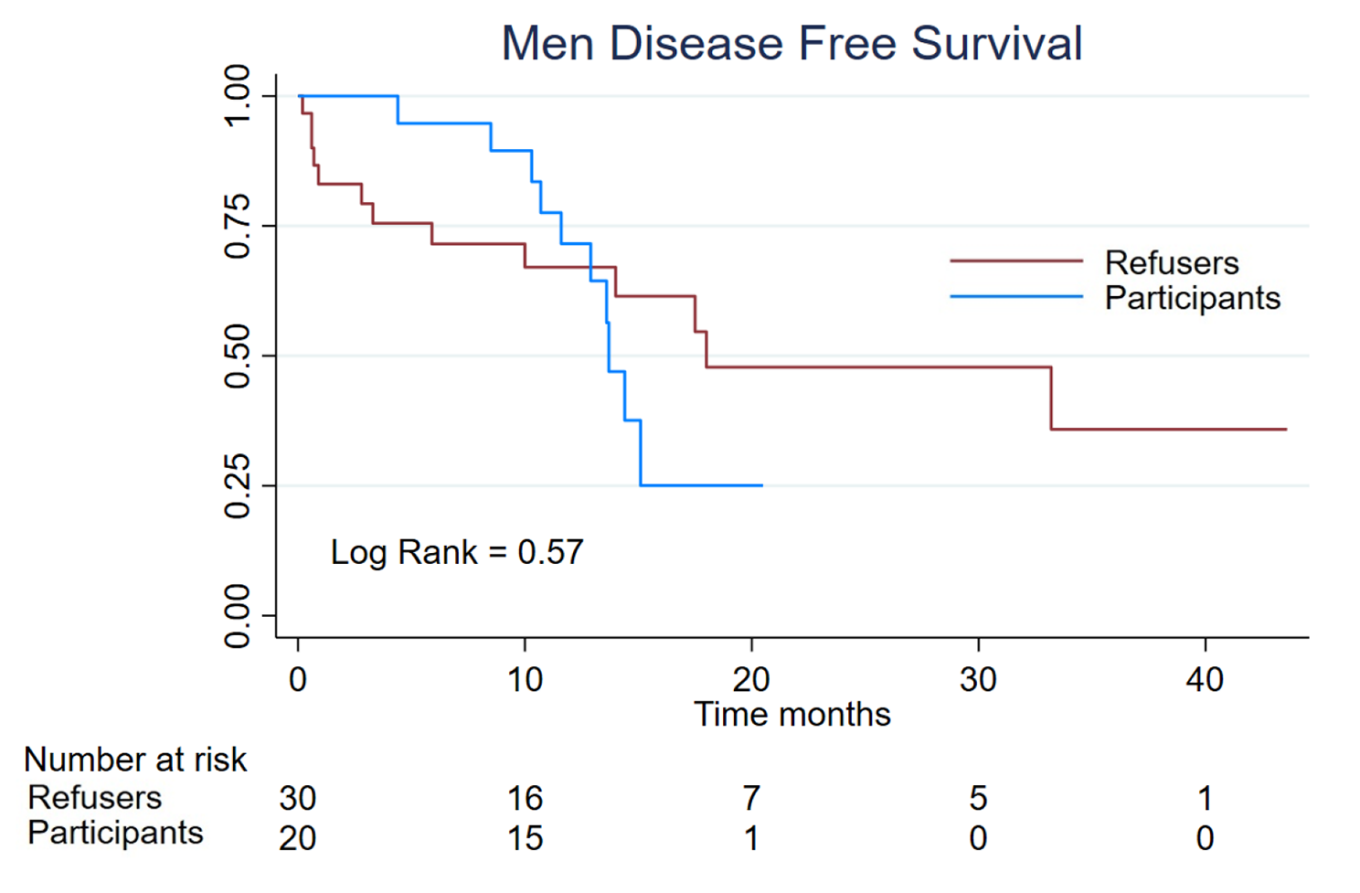
Men Disease Free Survival of 50 patients, divided into the participants (n = 20) and refusers (n = 30)

We identified 24 cases of progression, 7 (11,2%) in the women clinical trial participants and 17 (27,4%) in the refusers group; the median progression-free survival for refusers was 7,4 m (IC-95% 5,1–31,6) meantime for participants the median was not reached who 74,1% remained without progression at 28 month. The log-rank was 0,02 (Fig. 1). In the men group, we identified 23 cases of progression, 10 (20%) in the clinical trial participants and 13 (26%) in the refusers group; the median progression-free survival for participants was 13,7 m vs. 18 (Log-Rank 0,57) (Fig. 2).

### Overall survival

**Fig. 3:**
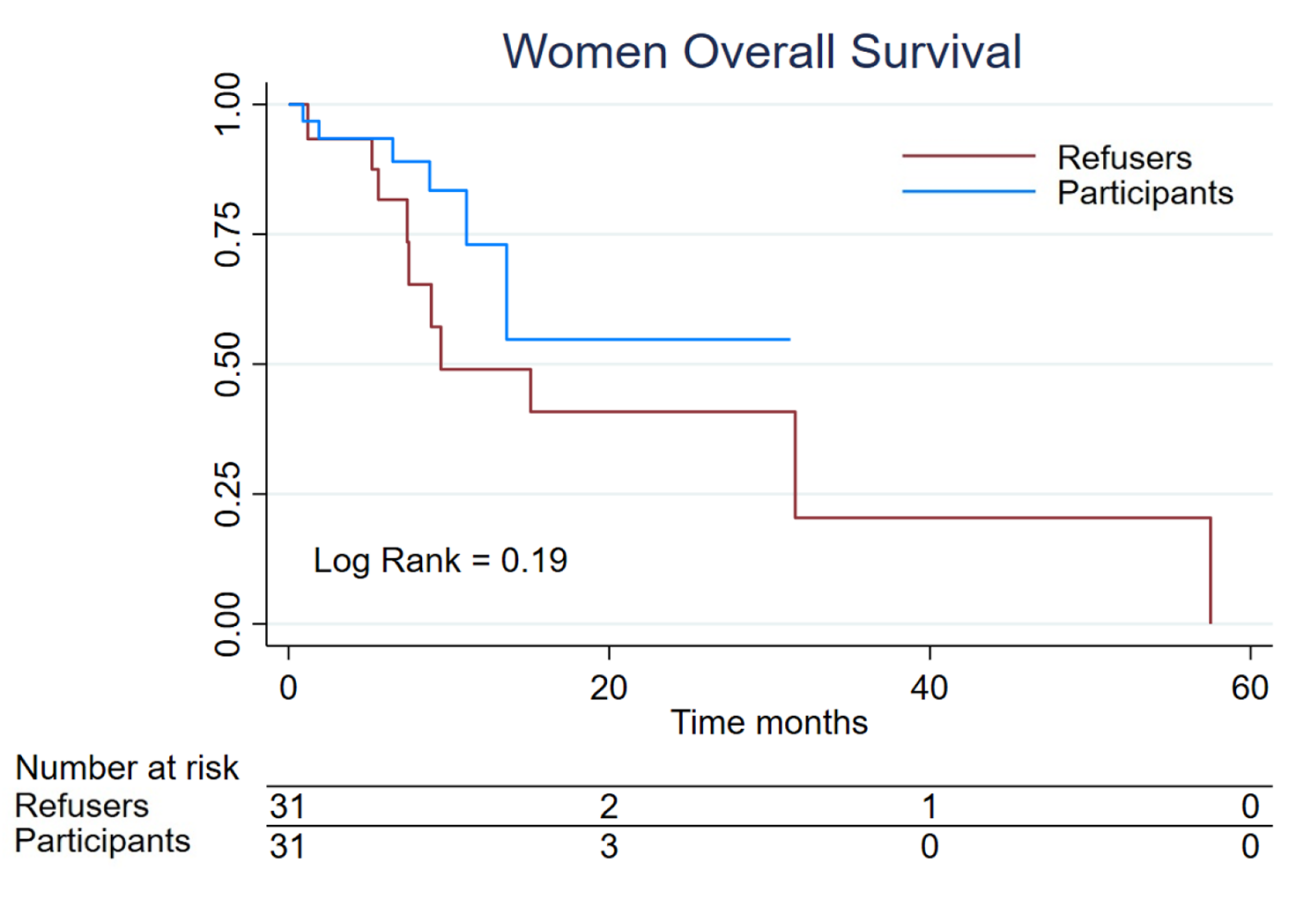
Women Overall Survival of 62 patients, divided into the participants (n = 31) and refusers (n = 31)

**Fig. 4:**
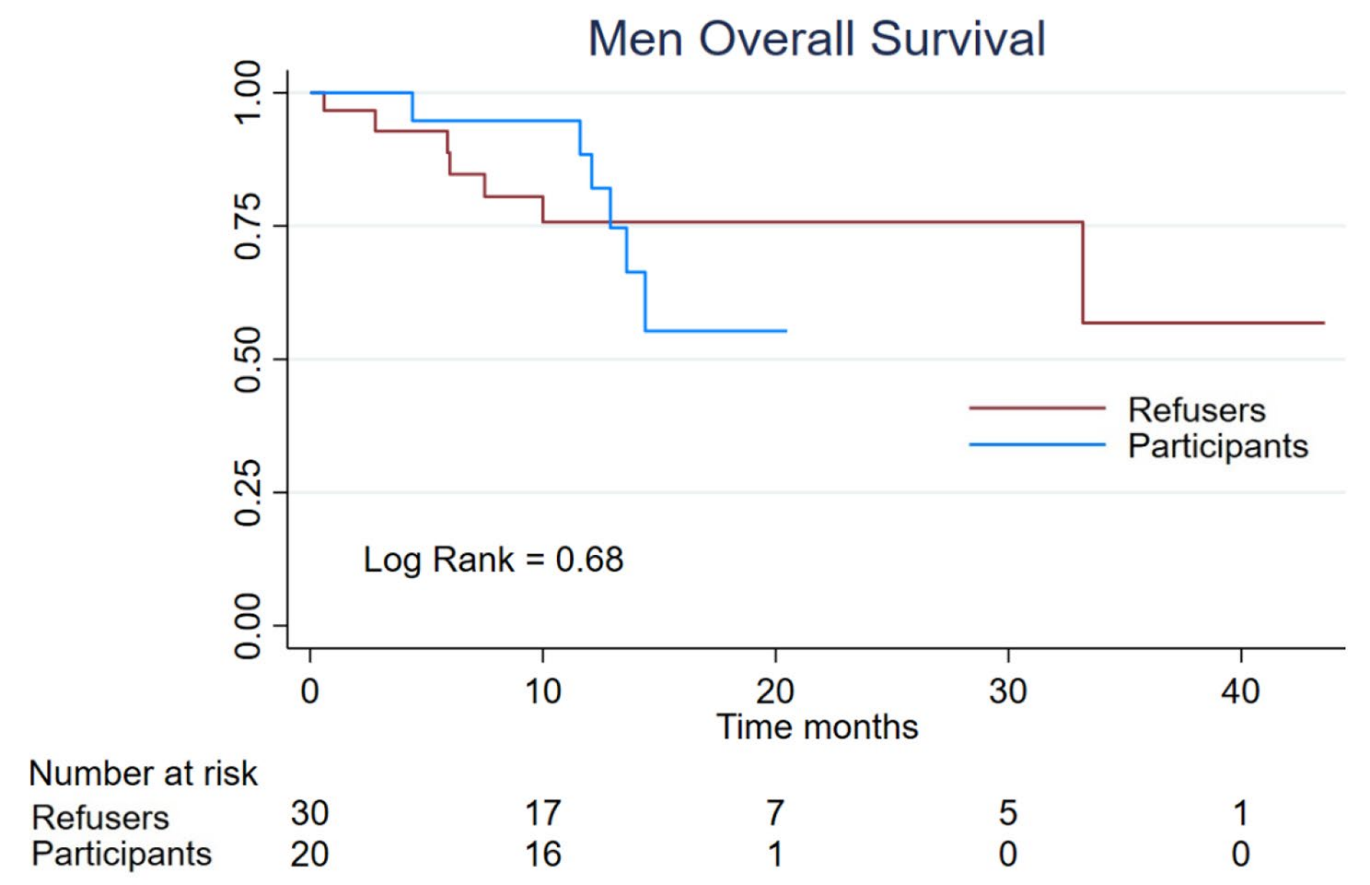
Men Overall Survival of 50 patients, divided into the participants (n = 20) and refusers (n = 30)

There were 6 (9,6%) deaths in the women clinical trial participants and 11 [7, 17] in the refusers group; the median survival for refusers was 15,1 m meantime for participants the median was not reached who 57,1% remained alive at 30 months. The log rank was 0,19 (Fig. 3). In the men group, we identified 13 deaths, 6 (12%) in the clinical trial participants and 7 (14%) in the refusers group. The median survival was not reached for any group, even though in the participants group 55,2% were still alive at month 20 and in the refusers group still alive at 56,8% at month 45 (log rank 0,68) (Fig. 4).

### Multivariate Cox regression análisis

Covariables included for the multivariate Cox regression were age, TMN, karnofsky, participant or refusers, but only age had a statistical association with overall survival in the women’s group HR 0,94 (IC-95% 0,90 − 0,99) p = 0,01 (Table 4). On the other hand in the men group any covariables reached statistical association (Table 5).

**Table 4:** Multivariate Cox regression analysis - Women

			HRs (95% CI)	*p* values
**Age (continuous)**			0,94 (0,90 − 0,99)	**0,018**
**Clinical T stage**			0,53 (0,27 − 1,07)	0,078
**Clinical N stage**			1,71 (0,62 − 4,65)	0,294
**Metastastic Disease**			3,57 (0,61 − 20,7)	0,155
**Karnofsky**			0,95 (0,88 − 1,02)	0,227
**Participants (yes vs. no)**			0,50 (0,17 − 1,44)	0,20

**Table 5:** Multivariate Cox regression analysis - Men

			HRs (95% CI)	*p* values
**Age (continuous)**			1,01 (0,97 − 1,05)	0,97
**Clinical T stage**			0,68 (0,35 − 1,33)	0,26
**Clinical N stage**			1,52 (0,65 − 3,55)	0,32
**Metastastic Disease**			1,17 (0,17 − 8,12)	0,17
**Karnofsky**			0,90 (0,79-1.02)	0,13
**Participants (yes vs. no)**			5,31 (0,99 − 28,7)	0,05

Therapies for the treatment of cancer are considered to be beneficial when they prolong survival, when they provide a palliative benefit in reducing symptoms, or when they improve the patient’s quality of life [4]. Accordingly, clinical trials seek to achieve an improvement in the efficacy outcomes with respect to standard therapy [13]. The efficacy of a new drug must be determined mainly by obtaining benefits on overall survival, disease-free and progression-free survival time, and to a lesser extent on surrogate outcomes such as the response rate to treatment and the behavior of some tumor markers [14][15].

Clinical trials must overcome barriers that limit their development; one of the main ones is the recruitment of patients which occurs in 77% and approximately 53% have to extend their duration and only 31% achieve the enrollment goals [16, 17]. It is estimated that only 3% of cancer patients manage to participate in clinical trials [18] and this low participation is mainly due to the lack of availability of certified hospitals for the development of clinical trials, the rigor in the enrollment of subjects which restricts admission to 3–5% of all subjects submitted to screening [19, 20] and by the refusal to participate of those candidate subjects [21, 22]. A local study established that 64% of patients would be willing to participate in a CT and that the most determinant factors in the decision are related to the information received about the risks and benefits, participation rights and informed, independent consent. Other factors such as sociocultural factors and education level [23] in this study accepted to participate in clinical trials was 45.5%. Many patients when asked to participate in clinical trials express high expectations and concerns that include, among others, the fear of presenting a reduction in their quality of life, the concern about receiving a placebo, the potential side effects, the concern that the drug in research may not be the best option, strictness of participation, aversion to randomization, feeling coerced, and loss of control over your treatment decisions [24–26].

The evidence supporting the belief that participation in clinical trials produces better clinical outcomes is insufficient [7], In this sense, the evidence that contributes to knowledge and allows adequately determining the impact of patient participation in clinical trials acquires high relevance. in cancer on clinical outcomes. Some published studies in this regard lack adequate comparability between participants and non-participants in a clinical trial, which is a fundamental factor that may call into question the generalization of the results because they include in their analyzes outcomes of regular care patients and patients enrolled in clinical studies [27].

In this research we sought to establish a better comparison between the groups, because from those patients who were eligible to participate in a clinical study, a comparison of clinical outcomes was made between those who agreed to participate and those who refused. It is important to note that both clinical trials groups included patients with advanced disease and the treatment included Pembrolizumab y placebo as type of control, but due to both trials being blinding we never knew who received intervention or control however never was not neccesary to open the blind in any trial.

We found subtle differences in the variables of social security, socioeconomic status, and clinical stage. Regarding clinical outcomes, a higher occurrence of non-serious adverse events was observed in the group of participants either men or women groups. however, this could be explained at least in part by the greater rigor in the recording of any adverse event during a clinical study than in real life clinical practice. It was also observed that the refuser group women presented more disease progression than the participants and mortality was higher in the group of refusers, similar to what was reported by Chow et al. to where participants in clinical trials had a lower risk of death [28]. Therefore, it can be considered that participation in clinical trials could give participants a better response to treatment, without increasing the probability of death and with the probability of decreasing the progression of the disease. participation in trials could improve the outcomes of clinical response rates, no change in overall survival, and progression-free.

Studies that have compared survival between participants have shown mixed results, while the Toxopeus study reported a median survival of 58.5 months (IQR 19.0-86.8) in the non-participant group vs. 35.0 (IQR 12.9–51.4). in the participant group (95% CI 16.1–29.4) [11]; in contrast to the study by Davis et al., it was reported that participation in a clinical trial was associated with better survival at 12 and 24 months 93% and 82% vs. 72% and 50% in non-participants [29]. In this study, we observed that the percentage of women participants alive at 24 months was 13,9% higher than refusers, on the other hand in the percentage of men participants alive at 24 months was 20,5% higher but in refusers, however making survival comparisons between these groups would not be adequate, due to large differences related to the selection criteria of patients who participate in clinical trials and patients of the standard of care they are not limited to these criteria.

## Conclusion

It continues to be complex to adequately establish survival patterns among participants in clinical trials; Factors such as age, the presence of comorbidities, the type of cancer, histology, clinical stage, functional status, type of treatment, among others, influence survival, especially in the first year, for which the rigor of the screening criteria Eligibility to enter a clinical trial that usually excludes patients with higher comorbidities could explain these differences [8].

Participants in clinical trials may experience better outcomes and longer survival rates than those receiving standard treatments but apart from the clinical outcomes, participating in a clinical trial can offer several advantages for cancer patients compared to those treated outside of clinical trials like to have access to cutting-edge treatments: Clinical trials often investigate new and innovative therapies that are not yet available. Participants in clinical trials may have access to novel drugs, targeted therapies, immunotherapies, or combination treatments that could potentially be more effective than standard treatments. Close monitoring and specialized care can lead to better management of side effects, early detection of complications, and personalized treatment adjustments. Contribution to medical knowledge: The data collected during the trial can help researchers and healthcare providers better understand the effectiveness and safety of new treatments. In some cases, clinical trial participants may receive the investigational treatment at no cost or with reduced expenses. Additionally, they might have access to additional support services that are not typically available in routine cancer care. It’s important to consider that clinical trials also come with potential risks and uncertainties. Experimental treatments may have unknown side effects or not yield the desired outcomes.

Finally, the results of this research should be taken with caution, considering the limitations of the design, the size of the sample, and the types of tumors that were included; however, these results may contribute to partially reducing the existing uncertainty about the effect of participation in clinical trials on some of the outcomes of cancer patients. Complementary studies are also required to help reduce the gap between the expectations of patients and the effect of participation on health status.

## Data Availability

Data is available in RedCap, but restrictions apply to the availability of these data. https://redcap.cancer.gov.co/redcap_v7.1.2/DataExport/index.php?pid=270.
